# Comparison of the efficacy of fully endoscopic spine surgery using transforaminal and interlaminar approaches in the treatment of prolapsed lumbar 4/5 disc herniation

**DOI:** 10.1186/s13018-022-03282-3

**Published:** 2022-08-13

**Authors:** Quanlai Zhao, Liang Xiao, Zhongxuan Wu, Chen Liu, Yu Zhang

**Affiliations:** Department of Spine Surgery, Yijishan Hospital, The First Affiliated Hospital of Wannan Medical College, 2 Zheshan West Road, Wuhu, Anhui 241001 People’s Republic of China

**Keywords:** Fully endoscopic spine surgery, Lumbar intervertebral disc herniation, Endoscopic transforaminal lumbar discectomy, Endoscopic interlaminar lumbar discectomy

## Abstract

**Background:**

There is still much controversy about whether transforaminal or interlaminar fully endoscopic spine surgery can better treat lumbar 4/5 disc herniation. Therefore, this study intends to compare the clinical efficacy of fully endoscopic spine surgery through transforaminal and interlaminar approaches in the treatment of lumbar 4/5 disc herniation.

**Methods:**

Seventy-six patients with lumbar 4/5 disc herniation admitted from March 2019 to June 2020 were divided into the transforaminal approach group (endoscopic transforaminal lumbar discectomy, ETLD; 54 cases) and the interlaminar approach group (endoscopic interlaminar lumbar discectomy, EILD; 22 cases) according to different surgical methods. The general clinical data and clinical evaluation scale of the patients were compared.

**Results:**

The post-operative ODI and VAS scores were significantly better in the EILD group (*P* < 0.05). The VAS and ODI scores of patients with upper-shoulder and sub-axillary types in the EILD group were superior to those in the ETLD group (*P* < 0.05), while the VAS and ODI scores of patients with the pre-radicular type were better when they underwent ETLD rather than EILD (*P* < 0.05). Patients with Lee zone III type in the EILD group had better post-operative ODI scores than those in the ETLD group (*P* < 0.05), but there was no significant difference in VAS scores (*P* > 0.05). Patients with Lee zone IV type who underwent EILD had better VAS and ODI scores than those who underwent ETLD (*P* < 0.05).

**Conclusions:**

For patients with a prolapsed intervertebral disc that belongs to the upper-shoulder type, sub-axillary type, or Lee III or IV type, EILD can achieve better outcomes.

## Background

As minimally invasive spinal techniques have advanced, fully endoscopic spine surgery (FESS) has received more attention worldwide due to its advantages of less bleeding, small operation wound, rapid post-operative recovery, and satisfactory efficacy in the treatment of lumbar disc herniation [1]. FESS is divided into the transforaminal-approach type (endoscopic transforaminal lumbar discectomy, ETLD) and interlaminar -approach types (endoscopic interlaminar lumbar discectomy, EILD) [2].

Currently, ETLD is mainly used to treat lumbar 4/5 intervertebral disc herniation, and EILD is mainly used to treat lumbar 5/sacral 1 intervertebral disc herniation. Reports on the analysis of the efficacy of the above two approaches are mostly limited to lumbar 5/sacral 1, while there are few studies on the treatment of intervertebral disc herniation of the lumbar 4/5 segments [3]. In addition, with the continuous development and innovation of FESS technology, EILD has also been gradually used for the treatment of intervertebral disc herniation of the lumbar 4/5 segments, but its clinical efficacy is still unclear.

In this study, we retrospectively analysed the efficacy and complications of ETLD and EILD in the treatment of intervertebral disc herniation of the lumbar 4/5 segments, aiming to provide a reference for selecting the appropriate surgical approach for clinical treatment of intervertebral disc herniation of the lumbar 4/5 segments.

## Patients and methods

### General information

The clinical data of 76 patients with intervertebral disc herniation of the lumbar 4/5 segments who underwent routine treatment in the Department of Spinal Orthopedics of our hospital from March 2019 to June 2020 were selected for analysis. The male:female ratio of the patients was 40:36. The age ranged from 15 to 89 years, with an average of 49.37 ± 14.05 years. The details of the general data are shown in Table 1.

**Table 1:** Patient demographic data

Parameter	Value
Follow-up time, months	20.37 ± 3.81
Sex ratio (M:F)	40:36
Age, years	49.37 ± 14.05
Course of disease, months	6.96 ± 4.17
The direction of herniation (left:right)	53:23
Operation time, mins	85.75 ± 22.60
Intraoperative fluoroscopy times (times)	6.00 ± 2.61
Post-operative hospital stay (days)	1.22 ± 0.53

**Fig. 1:**
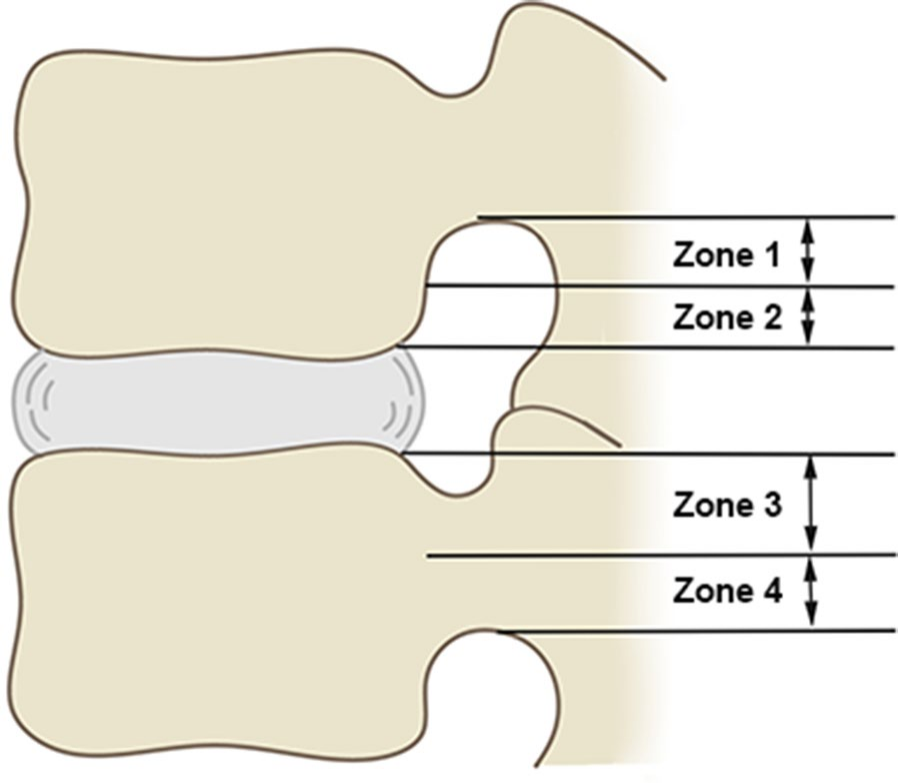
Lee partition of sagittal intervertebral disc prolapse

**Fig. 2:**
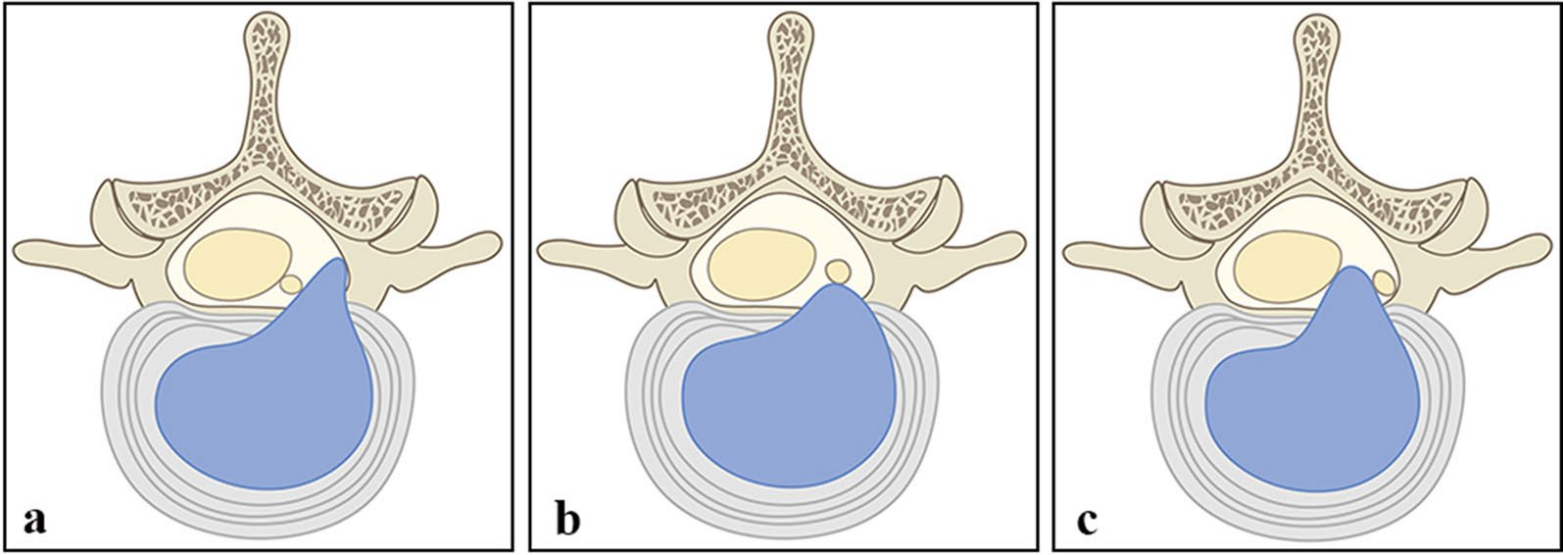
Division of transverse prolapse of intervertebral disc. (a) upper-shoulder type, **b** pre-radicular type, **c** sub-axillary type

All patients selected in this study mainly come from two time periods. The first was from March 2019 to March 2020. All patients with lumbar disc herniation in this time period were treated with ETLD. The second time period was from April 2020 to June 2020. All patients with lumbar disc herniation in this time period were treated with EILD. All patients were classified into zones I-IV according to the sagittal zoning method for intervertebral disc herniation by Lee et al. [4]. There were 56 cases of Lee zone III and 20 cases of Lee zone IV (Fig. 1). According to the relative position of the intervertebral disc herniation and nerve root [5] on the cross section, the patients were divided into 15 cases of upper-shoulder type, 36 cases of pre-radicular type, and 25 cases of sub-axillary type (Fig. 2). For detailed comparisons of the general information such as sex, age, and disease duration between the groups of patients treated with ETLD and EILD, see Table 2.

**Table 2:** Comparison of general data between ELLD and EPLD

	Number	Follow-up time (months)	Age (years)	Sex ratio (M: F)	Course of disease (months)	The direction of herniation (left:right)	Operation time (min)	Intraoperative fluoroscopy times	Post-operative hospital stay (days)
ELLD	54	20.81 ± 4.08	47.78 ± 13.96	31:23	6.74 ± 4.06	13:41	89.09 ± 21.83	7.33 ± 1.64	1.17 ± 0.37
EPLD	22	19.27 ± 2.83	53.27 ± 13.84	9:13	7.50 ± 4.46	10:12	77.55 ± 22.86	2.73 ± 1.31	1.36 ± 0.79
*t*		1.617	1.560	1.707	0.717	3.386	2.063	12.823	1.120
*P*		0.110	0.123	0.191	0.475	0.066	0.043	0.000	0.274

### Surgical procedures

#### ETLD

**Fig. 3:**
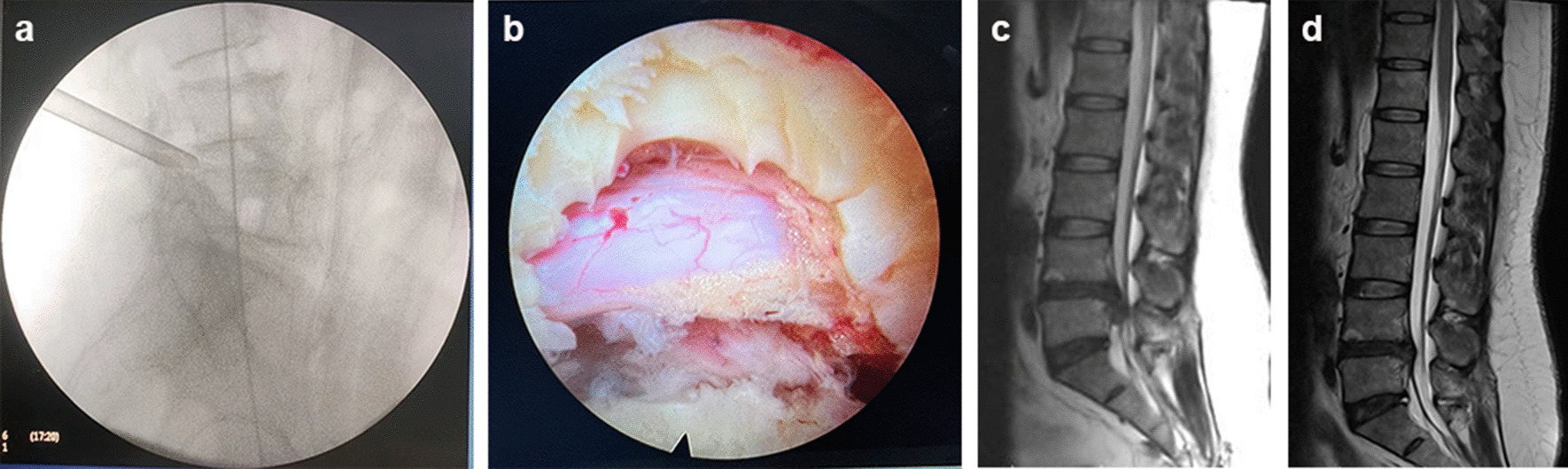
ETLD. **a** Preoperative fluoroscopy localization, **b** macroscopic observation of nerve roots under endoscope after operation, **c** preoperative MRI sagittal image, **d** post-operative MRI sagittal image

The patients underwent local anaesthesia (lidocaine with normal saline, 1:1) and were given intravenous adjuvant drugs (dexmedetomidine, 0.5-1ug/kg/h). With the patient in the jackknife position, the ipsilateral iliac crest line, midline of the spinous process, and responsible disc space were marked. After routine disinfection and draping, the puncture needle was inserted at 8–10 cm away from the midline of the spinous process with a 5–10° angle to the horizontal line of the intervertebral space towards the head. The puncture needle sequentially penetrated the locally anaesthetized skin, subcutaneous tissue, deep fascia, and muscle until reaching the ventral side of the articular process of lumbar 5. After the puncture needle was in place, we made an 8-mm incision on the skin and expanded the soft tissue using a gradually expanding cannula. A visual trephine was used to remove part of the bone on the ventral side of the superior articular process to enlarge the area of the intervertebral foramen. After a satisfactory visual field was achieved, a working cannula was inserted to remove the dorsal ligamentum flavum tissue, expose the nerve root, and enter the ventral side of the nerve root. The prolapsed intervertebral disc tissue was removed with nucleus pulposus forceps, and radiofrequency was used to fully stop bleeding (Fig. 3).

#### EILD

**Fig. 4:**
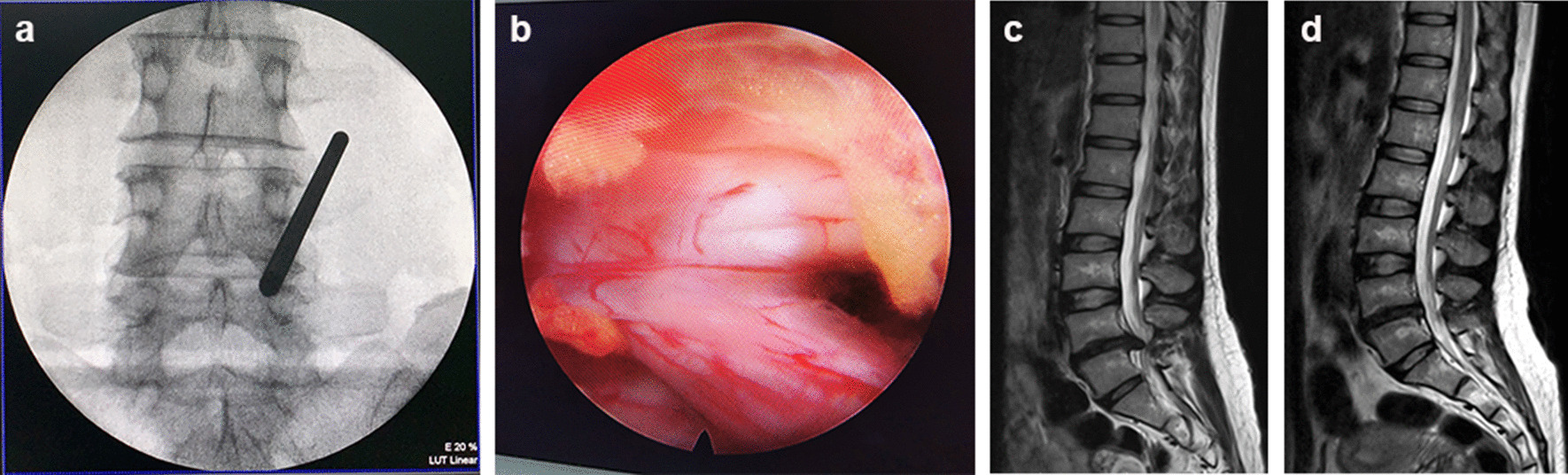
EILD. **a** Preoperative fluoroscopy localization, **b** macroscopic observation of nerve root under endoscope after operation, **c** preoperative MRI sagittal image, **d** post-operative MRI sagittal image

The patients underwent general anaesthesia or spinal anaesthesia and took the jackknife position. Routine disinfection and draping were performed, and an incision with the length of approximately 1 cm was made on the skin at the intersection point of the line between the inner edge of adjacent upper and lower pedicles on the operated side and the horizontal line of the intervertebral space. After the skin and subcutaneous fascia were incised with a sharp knife, the visual trephine cannula was placed through the dilator, with its opening facing the upper vertebral lamina. The soft tissue was cleaned to fully expose the upper and lower vertebral laminae and ligamentum flavum fossa under the endoscope, and then the upper and lower vertebral laminae were opened in a “U” shape with a visual trephine in the counterclockwise direction under the endoscope to expose the upper, lower, and lateral stop points of the ligamentum flavum on the operated side. Under the endoscope, the medial margin of the superior articular process was treated with a bone rongeur to expose the lamina fenestration. A microscopic nerve stripper was used to separate the surrounding adhesive tissue along the outer edge of the nerve root. The nerve root was pushed inward and the outer working cannula was pushed into the spinal canal to reach the outside of the nerve root. The outer working cannula was moved up and down, inside and outside, to look for the rupture of the annulus fibrosus and remove the prolapsed intervertebral disc tissue (Fig. 4).

### Perioperative treatment

Relevant examinations were done before surgery to rule out surgical contraindications. For 6–12 h after the operation, the patient wore a hard waist brace to get out of bed under the guidance of a physician. The patient was discharged 1 days after the operation and returned to normal work and home life 6 weeks later. All patients were followed up for 12 to 27 months, with an average of 20.37 ± 3.81 months. The patients were evaluated by Oswestry Disability Index (ODI) and visual analogue scale (VAS) scores before the surgery and at the last follow-up after surgery. All patients had no complications or reoperation during post-operative follow-up.

### Statistical analysis

SPSS 18.0 (SPSS, USA) statistical software was used for statistical analysis. All measurement data were tested for normal distribution characteristics. Measurement data were compared between the two groups by the independent-sample t test, and count data were compared by the *χ*^2^ test. *P* < 0.05 was considered statistically significant.

## Results

### The clinical efficacy of EILD is better than that of ETLD

The preoperative ODI score was 71.37 ± 5.23 and 71.73 ± 5.49 for the ETLD group and EILD group, respectively, and the VAS score was 6.06 ± 0.73 and 6.09 ± 0.68 (*P* > 0.05). The post-operative ODI score of the patients in the ETLD group was 7.81 ± 2.17 and post-operative VAS score 1.87 ± 0.72, while the post-operative ODI score of the patients in the EILD group was 5.73 ± 2.07 and their post-operative VAS score 1.50 ± 0.59 (both *P* < 0.05). See Table 3 for details.

**Table 3:** Comparison of VAS and ODI scores between patients with ELLD and EPLD before and after operation

	Number	Preoperative ODI (%)	Preoperative VAS (points)	Post-operative ODI (%)	Post-operative VAS (points)
ELLD	54	71.37 ± 5.23	6.06 ± 0.73	7.81 ± 2.17	1.87 ± 0.72
EPLD	22	71.73 ± 5.49	6.09 ± 0.68	5.73 ± 2.07	1.50 ± 0.59
*t*		0.266	0.193	3.848	2.111
*P*		0.791	0.847	0.000	0.038

### The efficacy of EILD for the upper-shoulder and sub-axillary type is superior to that ETLD

There were 25 patients with sub-axillary type, nine of whom underwent ETLD, 16 EILD. The preoperative ODI score was 71.11 ± 5.92 and 72.63 ± 5.73, and the VAS score was 6.11 ± 0.60 and 6.19 ± 0.65 in the sub-axillary ETLD and sub-axillary EILD group, respectively (*P* > 0.05); the post-operative ODI score was 8.67 ± 1.73 and the post-operative VAS score 2.33 ± 0.86 in the sub-axillary ETLD patients, while the post-operative ODI score was 5.63 ± 2.21 and the post-operative VAS score 1.56 ± 0.62 in the sub-axillary EILD patients (*P* < 0.05). See Table 4 for details.

**Table 4:** Comparison of VAS and ODI scores between patients with sub-axillary type before and after operation (n = 25)

	Number	Preoperative ODI (%)	Preoperative VAS (points)	Post-operative ODI (%)	Post-operative VAS (points)
ELLD	9	71.11 ± 5.92	6.11 ± 0.60	8.67 ± 1.73	2.33 ± 0.86
EPLD	16	72.63 ± 5.73	6.19 ± 0.65	5.63 ± 2.21	1.56 ± 0.62
*t*		0.626	0.288	3.541	2.568
*P*		0.537	0.776	0.002	0.017

There were 36 patients with the pre-radicular type underwent ETLD. The preoperative ODI score was 71.50 ± 5.24 and the post-operative ODI score was 7.50 ± 2.26 (*P* < 0.05). The preoperative VAS score was 6.17 ± 0.77 and the post-operative score was 1.67 ± 0.63 (*P* < 0.05). See Table 5 for details.

**Table 5:** Comparison of VAS and ODI scores between patients with pre-radicular type before and after ELLD (*n* = 36)

	Number	Preoperative	Post-operative	paired t	*P*
ODI (%)	36	71.50 ± 5.24	7.50 ± 2.26	72.718	0.000
VAS (points)	36	6.17 ± 0.77	1.67 ± 0.63	24.946	0.000

There were 15 patients with the upper-shoulder type, 9 of whom underwent ETLD, 6 EILD. The preoperative ODI score was 71.11 ± 5.11 vs. 69.33 ± 4.32, and the VAS score was 5.56 ± 0.52 vs. 5.83 ± 0.75 in the upper-shoulder ETLD vs. upper-shoulder EILD group, respectively (*P* > 0.05). The post-operative ODI score was 8.22 ± 2.10and the post-operative VAS score was 2.22 ± 0.66 in upper-shoulder ETLD patients, and these numbers were 6.00 ± 1.78(*P* > 0.05) and 1.33 ± 0.51(*P* < 0.05) in the upper-shoulder EILD patients. See Table 6 for details.

**Table 6:** Comparison of VAS and ODI scores between patients with upper-shoulder type before and after operation (*n* = 15)

	Number	Preoperative ODI (%)	Preoperative VAS (points)	Post-operative ODI (%)	Post-operative VAS (points)
ELLD	9	71.11 ± 5.11	5.56 ± 0.52	8.22 ± 2.10	2.22 ± 0.66
EPLD	6	69.33 ± 4.32	5.83 ± 0.75	6.00 ± 1.78	1.33 ± 0.51
*t*		0.700	0.845	2.117	2.750
*P*		0.497	0.413	0.054	0.017

### The efficacy of EILD in patients with Lee zone IV is superior than that of ETLD

There were 56 patients with Lee zone III, 47 of whom underwent ETLD, 7 EILD. The preoperative ODI score was 71.43 ± 5.08 vs. 72.29 ± 5.58, and the VAS score was 6.12 ± 0.72 vs. 6.29 ± 0.48 in Lee zone III ETLD group vs. Lee zone III EILD group, respectively (*P* > 0.05). The post-operative ODI score was 7.76 ± 2.22 in the Lee zone III ETLD group and 4.86 ± 1.06 in the EILD group (*P* < 0.05). The post-operative VAS score was 1.80 ± 0.70 in the Lee zone III ETLD group and 1.86 ± 0.69 in the EILD group (*P* > 0.05). See Table 7 for details.

**Table 7:** Comparison of VAS and ODI scores between patients with Lee zone III type before and after operation (n = 56)

	Number	Preoperative ODI (%)	Preoperative VAS (points)	Post-operative ODI (%)	Post-operative VAS (points)
ELLD	49	71.43 ± 5.08	6.12 ± 0.72	7.76 ± 2.22	1.80 ± 0.70
EPLD	7	72.29 ± 5.58	6.29 ± 0.48	4.86 ± 1.06	1.86 ± 0.69
*t*		0.413	0.575	3.375	0.215
*P*		0.682	0.568	0.001	0.831

There were 20 patients with Lee zone IV, 5 of whom underwent ETLD, 15 EILD. The preoperative ODI score was 70.80 ± 7.29 vs. 71.47 ± 5.63 and the VAS score was 5.40 ± 0.54 vs. 6.00 ± 0.75 in the Lee zone IV ETLD group vs. Lee zone IV EILD group, respectively (*P* > 0.05). The post-operative ODI score was 8.40 ± 1.67 and the post-operative VAS score was 2.60 ± 0.54 in the Lee zone IV ETLD group, while the post-operative ODI score was 5.60 ± 1.72 and the post-operative VAS score was 1.33 ± 0.48 in the Lee zone IV EILD group (*P* < 0.05). See Table 8 for details.

**Table 8:** Comparison of VAS and ODI scores between patients with Lee zone IV type before and after operation (n = 20)

	Number	Preoperative ODI (%)	Preoperative VAS (points)	Post-operative ODI (%)	Post-operative VAS (points)
ELLD	5	70.80 ± 7.29	5.40 ± 0.54	8.40 ± 1.67	2.60 ± 0.54
EPLD	15	71.47 ± 5.63	6.00 ± 0.75	5.60 ± 1.72	1.33 ± 0.48
*t*		0.214	1.625	3.166	4.888
*P*		0.833	0.121	0.005	0.000

## Discussion

In recent years, with the continuous development and improvement in minimally invasive concepts in spinal surgery, the application of visualization technology to the treatment of lumbar disc herniation has gradually become more widespread. FESS not only greatly improves surgical efficiency and safety but also minimizes harmful radiation exposure to surgeons and patients compared with previous blinded operations [1]. At present, the endoscopic techniques applied to lumbar disc herniation have their own advantages and disadvantages, though the most widely used are lateral-approach FESS and posterior-approach FESS [6, 7]. The transforaminal approach mainly uses the ventral side of the facet as the starting point under the endoscope, sawing towards the proximal end to enlarge the area of the foramen, so that the trephine cannula can fit inside and is closely attached to the base of the facet and the pedicle. As a result, the nerve root and prolapsed disc tissue are exposed. This surgical procedure tends to rely on the puncture feeling of the surgeon and has a relatively lower work efficiency. Particularly for novices, there is often a possibility of insufficient decompression and disc removal failure. In addition, intraoperative complications such as damage to blood vessels and nerves also occur frequently [8]. The interlaminar approach surgery mainly performs vertebral lamina fenestration and nucleus pulposus removal. The application of visual trephine can perform "U"-shaped expansion of the vertebral lamina in a clockwise direction to fully expose the upper, lower, and lateral stop points of the ligamentum flavum, which avoids the pushing and pulling of the nerve that occur in transforaminal approach surgery. There is still much controversy about whether transforaminal or interlaminar FESS can better treat lumbar disc herniation [9, 10].

In this study, we retrospectively compared the difference in the clinical efficacy between ETLD and EILD in the treatment of lumbar 4/5 disc herniation. The results showed that both ETLD and EILD significantly relieved the symptoms of low back and leg pain, with no significant difference in the post-operative hospital stay or recurrence rate. ETLD has a longer operation time, more fluoroscopies, and higher incidence of residuals than EILD, while EILD has more post-operative skin paraesthesia and a higher probability of nerve injury in relevant reports, which is mainly due to cannula misplacement or interference with ligamentum flavum identification by structures such as facet joint cysts, muscles, and ligaments [11–13].

To further explore the differences in the treatment of disc herniation between the above two surgical methods, we divided and compared the 76 included patients according to the position of the prolapsed intervertebral disc in the cross section and sagittal section. Specifically, in the sagittal plane, grouping was performed according to the zones proposed by Lee et al. Zones I and II type were upward herniation of the disc, and zones III and IV type were downward herniation. In the transverse plane, according to the relative position of the intervertebral disc and nerve roots, they were divided into the sub-axillary type, pre-radicular type, and upper-shoulder type. Since the volume of the spinal canal decreases as it moves up, patients with Lee zone I type are rarely found, and only four patients belong to Lee zone II type in this study, so they were not studied. Comparison of the 56 patients with Lee zone III type revealed that EILD patients yielded better post-operative ODI scores than ETLD, with no difference in VAS scores. Comparison of the 20 patients with Lee zone IV type revealed that the post-operative ODI and VAS scores of the EILD patients were superior to those of the ETLD patients. We believe that this may have been due to the excessive downward herniation of the intervertebral disc. When ETLD is performed, it requires more work for facetoplasty and pediculoplasty, and the procedure is long. If the prolapsed intervertebral disc is not one piece, it is difficult to remove completely. On the other hand, prolonged cannula placement for the removal will inevitably disturb the nerve roots and affects the post-operative outcome [14]. Although many scholars have tried to improve ETLD, these approaches are technically demanding and currently have no standardized operating procedure and are not reproducible [15, 16]. Therefore, EILD is more effective than ETLD for patients with downward herniation of a disc, especially patients with Lee zone IV type.

EILD can also achieve better surgical outcomes than ETLD for patients with upper-shoulder and sub-axillary type. For patients with the upper-shoulder type, the intervertebral disc is often less prolapsed, and most of these patients belong to Lee zone III type. For patients with the sub-axillary type, the intervertebral disc is often more prolapsed, and most of these patients belong to Lee zone IV type at the same time. In the upper-shoulder type, the prolapsed intervertebral disc is hidden at the medial edge of the pedicle and forms a 90° angle with the working cannula, which is in the blind area of the field of view, so cryptoplasty and pediculoplasty are required in ETLD. However, this kind of pediculoplasty is technically difficult and prone to bleeding or damage of the pedicle. In addition, once the prolapsed disc is broken into multiple fragments, it is highly prone to residual and incomplete removal in ETLD [17, 18]. EILD can better visually detect the intervertebral disc and enable removal, with a shorter operation time and less nerve damage. In the sub-axillary type, the intervertebral disc prolapse is relatively distant. When ETLD is performed for the removal, the prolapsed intervertebral disc needs to be pulled from the medial side to the lateral side of the nerve root in the form of "fishing." In this process, it is extremely easy to damage the nerve root, and patients often insist on interrupting the operation due to intolerable pain [19]. It is worth noting that in the sub-axillary type, there may be some retraction or strain in the intervertebral disc removal through EILD, and the patient cannot timely report the nerve discomfort to the doctor because of the general anaesthesia. Therefore, doctors should carefully study the imaging data before operation and be careful during operation [20–23]. The prolapse of the intervertebral disc is often greater in the pre-radicular type, and because the intervertebral disc is located in front of the nerve root, the nerve root and dura mater are often squeezed to the dorsal side. At this time, it is not easy to find the intervertebral disc by using EILD, and it is necessary to push the nerve root to remove the intervertebral disc. Therefore, ETLD is more advantageous in theory. However, the pre-radicular type patients selected in this study all used ETLD, so it was impossible to compare the prognosis difference between ETLD and EILD. There were some limitations in this study, including the small number of cases included, and the classification of intervertebral disc types was not comprehensive enough.

## Conclusion

Overall, for lumbar 4/5 disc herniation, although both ETLD and EILD have good surgical outcomes, for patients with Lee zone III or IV type and a upper-shoulder or sub-axillary type, EILD can achieve better efficacy.

## Data Availability

The datasets used and/or analysed during the current study are available from the corresponding author on reasonable request.

## Abbreviations

FESS: Fully endoscopic spine surgery
ETLD: Endoscopic transforaminal lumbar discectomy
EILD: Endoscopic interlaminar lumbar discectomy
ODI: Oswestry disability index
VAS: Visual analogue scale
